# Supplementary material for: Good vibrations: Sternal vibration enhances white matter density and interoceptive awareness
Source: Neuropsychopharmacology. 2026 May 16;51(8):1369–77. doi: 10.1038/s41386-026-02430-1 (PMC13291253; doi:10.1038/s41386-026-02430-1)
Supplement: Supplementary file 1 — Supplement [file 41386_2026_2430_MOESM1_ESM.docx]

**Supplemental Material**

**Study Attrition and Reasons for Dropout**

As of October 15^th^, 2024, a total of 262 participants were consented to the trial, and 93 of these participants did not start a study intervention (e.g., changes in availability, non-completion of MRI). 169 participants were randomized and did start a study intervention, and there were 157 participants who completed the full intervention protocol. 11 dropped out during the intervention due to loss of interest or changes in their availability, and 1 participant was excluded during the intervention due to an incidental discovery of a tumor on their pre-intervention MRI scan (not counted toward retention rates). Overall, retention rates for the trial have been high with 93.5% of participants who completed their pre-scan and at least one intervention session eventually finishing the full protocol (157/168). Retention rates were marginally higher (*χ²*=2.97, *p*=0.085) for those who received vibration (96.6%; 85/88) compared to those who did not (90.0%; 72/80). There were 30 additional participants who completed the intervention but were not able to complete both pre/post dMRI scans due to scanner time constraints (*n*=30; dMRI was the final run of a lengthy scanning protocol). Thus, of the 157 participants who completed the full intervention protocol, there were 127 participants had pre/post dMRI data. However, 11 of these participants’ data were excluded due to quality concerns (e.g., low total signal to noise ratio), which led to a final sample of 116 participants with useable pre/post dMRI data. Also, tractography processing failed for 2 subjects, who were excluded from those analyses only. The CONSORT diagram is depicted in Table S1.

**Covariate Adaptive Randomization Process Details**

Covariate adaptive randomization (Lebowitsch et al., 2012) is an unbalanced minimization method also referred to as the multidimensional dynamic allocation method. This method takes into account that new subjects coming into the study and assigns a treatment group based on the subjects already assigned to a treatment group. It seeks to minimize the amount of unbalance between groups for the designated variables simultaneously. This minimization occurs across levels, including the strata, factors, and overall study. The relative weight for each level is decided upon by the investigators depending on the study design. This method can be applied to any number of treatment arms. In the present study, covariate adaptive randomization to randomize subjects was based on biological sex assigned at birth, race, ethnicity, age group, and baseline dissociation severity (multiscale dissociation inventory score).

**Detailed Intervention Procedures**

Participants were randomized to one of 4 conditions that were either a vibration (VABF, pulsed vibration) or non-vibration (breath-focused mindfulness, open awareness) condition. Participants remained in the same intervention and received the same instructions for all eight visits. Participants completed approximately 1–3 visits weekly over 4–8 weeks depending on their availability. This flexibility in spacing was designed to enhance study retention while maintaining protocol fidelity. During each intervention visit, participants received brief instructions related to their respective intervention condition via computer monitor. Irrespective of the randomized intervention condition, participants wore a low frequency haptic transducer on the chest and a pneumatic respiration cushion to measure breathing. Intervention visits consisted of six, three-minute blocks of mindfulness meditation, consistent with the assigned intervention’s instructions (18 min total). With set-up and debriefing, visits lasted ∼45–75 min. Participants randomized to VABF were instructed to “Please focus on your breathing and the associated vibration you feel on your chest” during each intervention visit. Vibration [breath-like vibration patterns (sharper rise followed by prolonged fall in magnitude of a ∼100 Hz signal) lasting ∼6 seconds; synchronized to the start of exhalation in VABF and triggered every 6 seconds in pulsed vibration] came from the haptic transducer, an experimental prototype. VABF feedback was provided based on participant’s own respiration patterns measured using customized Matlab routines. Participants in the ‘breath-focus only’ condition were instructed to “Please focus on your breathing” during each intervention visit. Participants randomized to ‘vibration only’ were instructed to “Please stay aware as you feel vibration on your chest” during each intervention visit. Participants randomized to ‘open awareness’ were instructed to “Please allow yourself to be open to your experiences” during each intervention visit. Study conditions were collapsed into ‘vibration’ (combined VABF and vibration only conditions) and ‘non-vibration’ (combined breath focus and open awareness conditions) conditions for analyses.

**Imaging Acquisition and Processing Details**

*MRI Acquisition and Image Processing*. MRI scans were acquired on two research-dedicated Siemens 3-Tesla Prisma^fit^ MRI systems, one at each site. Multi-shell diffusion weighted imaging (DWI) was obtained with optimal angular coverage using 128 diffusion directions distributed over 4 shells (4 volumes of b = 300 s/mm^2^, 17 of b = 650, 39 of b = 1,000, and 68 volumes of b = 2,000 s/mm^2^), with 2 mm^3^ isotropic voxel resolution, multiband factor 3, TE = 79 ms, TR = 2,750 ms, flip angle = 78°, AP phase encode (PE) direction = AP, and 232 × 256 FOV. Additionally, 12 b = 0 s/mm^2^ images were acquired interspersed between the diffusion volumes. We also acquired two volumes of b = 0 s/mm^2^ in the opposite PE direction to correct for distortion and other artifacts. Additionally, a 3D-T1 MPRAGE with 0.8mm^3^ isotropic resolution was acquired for co-registration with diffusion images. All diffusion-weighted image processing and analysis were conducted using FMRIB Software Library [FSL version 4.1; www.fmrib.ax.ac.uk/fsl; ^34^].

*Diffusion-weighted Image Processing.* dMRI images were corrected for susceptibility-induced distortion, eddy currents and subject motion using ‘topup’ and ‘eddy’ tools in FSL. A brain mask was generated from averaged and corrected b=0 images using the FSL’s brain extraction tool (BET)^35^. Fractional anisotropy (FA) maps were generated using the ‘DTIfit’ in the FMRIB Diffusion Toolbox. Multiple compartment modeling was performed on diffusion-weighted data using NODDI^36,37^. To do so, a GPU-enabled CUDA diffusion modelling toolbox (cudiMOT) was used to generate Bingham-NODDI maps^36^, including Neurite Density Index (NDI) and Orientation Dispersion Index (ODI). Compared to the conventional Watson-NODDI model, which assumes isotropic dispersion of neurites^37^, Bingham-NODDI provides a better estimation of the anisotropic orientation dispersion commonly encountered within regions of fanning and bending^36^. Formal quality checks were performed on the data by calculating the temporal signal-to-noise ratio (tSNR) across each diffusion volume using ‘eddy_qc’ tools in FSL^38^. To account for differences in scanner and scan quality via tSNR, scalar maps were harmonized using ComBat-GAM, now referred to as neuroHarmonize^39^.

*Voxel-wise Analyses of Intervention Change*. Voxel-wise differences in NODDI scalar indices were assessed using Tract-based Spatial Statistics (TBSS, version 1.2, available in FSL), an approach that increases the sensitivity and interpretability of the results compared with typical voxel-based approaches because it uses non-linear registration^40^. All participants’ FA maps were co-registered using the non-linear registration to the most ‘typical’ participant's FA [as determined by FMRIB's nonlinear image registration tool^41^], then affine transformed into 1 × 1 × 1 mm MNI space. All transformed FA images were averaged to create a mean FA image, then thresholded by FA > 0.2 to ensure gray matter regions would be excluded from these analyses. The mean FA skeleton was also used to project non-FA maps (NDI and ODI) into study specific metric skeletons, using the same transformations and projection parameters as applied to the FA maps.

*Replication Analyses with Probabilistic Tractography*. Tracts that demonstrated significant time and/or intervention-related changes in primary voxel-wise analyses were reconstructed using probabilistic tractography to assess for replication. Markov Chain Monte Carlo sampling was used to calculate within-voxel probability density functions of the principal diffusion direction using FSL’s Bayesian Estimation of Diffusion Parameters Obtained using Sampling Techniques (BEDPOSTX) tool^42,43^, which also accounts for the possibility of crossing fibers within a voxel. Probabilistic fiber tracking was conducted with PROBTRACKX implemented in FSL; this method repeatedly samples the distribution at each voxel to produce “streamlines” that connect voxels from selected seed regions (5,000 streamline samples, .5 mm step length, curvature threshold = .2). Probabilistic tractography was conducted using XTRACT, a standardized and automated tractography tool implemented in FSL^44-46^. XTRACT was used to reconstruct 42 major white matter tracts using pre-defined, anatomically-constrained seed, target, exclusion, and stop masks in subject’s diffusion space. Mean NODDI metrics were then extracted from the tracts of interest.

**Supplemental Figure and Tables**

Following the supplemental references, provided below are a supplemental figure and tables. Figure S1 depicts the CONSORT diagram for the present study and details reasoning behind participant inclusion/exclusion and provides detailed information regarding reasons for non-completion. Figure S2 depicts the moderating effect of all four intervention conditions on the association between left cerebral peduncle NDI changes and changes in SBC: Body Dissociation ratings (effect was driven by sternal vibration). Table S1 provides information regarding the medications and psychiatric diagnoses for each group and compares frequency counts using Pearson’s χ^2^ tests. A full description of medication categories is provided below. Table S2 reports full results for tractography analyses that replicated tract-based spatial statistics findings. Table S3 reports results from analyses of self-reported changes in Scale of Body Connectedness scores. Table S4 reports additional confirmatory ANOVAs testing with covariates of baseline PTSD symptom severity, current PTSD diagnosis, depression symptom severity, current major depressive episode, prior experience with mindfulness meditation, and days between final intervention session and post-scan all still showed significant time-by-vibration interaction effects on the left and right cerebrospinal tract (CST).

For medication categories, descriptors and examples included were: *Antidepressant/SSRI/SNRI* (Zoloft, Prozac, Trazodone, Cymbalta/Duloxetine, Wellbutrin, Celexa/Citalopram, Mirtazapine/Remeron, etc.); *Anticonvulsant/Mood Stabilizer* (Neurontin, Gabapentin, Depakote, Lamictal, Topamaz, Lyrica); *Antipsychotic* (Seroquel/Quetiapine, Abilify/Aripiprazole); *Benzodiazepine* (Clonazepam/Klonopin, Xanax, Lorazepam/Ativan, Diazepam/Valium); *Tricyclic* (Doxepin, Amitriptyline, etc.); *Serotonergic Receptors* (Maxalt, migraine medications, etc.); *Stimulant* (Vyvanse, Concerta); *Muscle Relaxant* (Flexeril); *Hypnotics* (Ambien, Lunesta, sleep aid, etc.); *Cardiovascular* (Alpha & Beta Blockers, Lisinopril, Losartan, Labetalol, Propranolol, Amlodipine, Hydrochlorothiazide, diuretic, rosauvastatin, imdur); *Endocrine* (Birth control, steroids, etc.); *Thyroid agents* (Synthyroid, Levothyroxine).

**Figure S1.** *CONSORT Diagram*

**
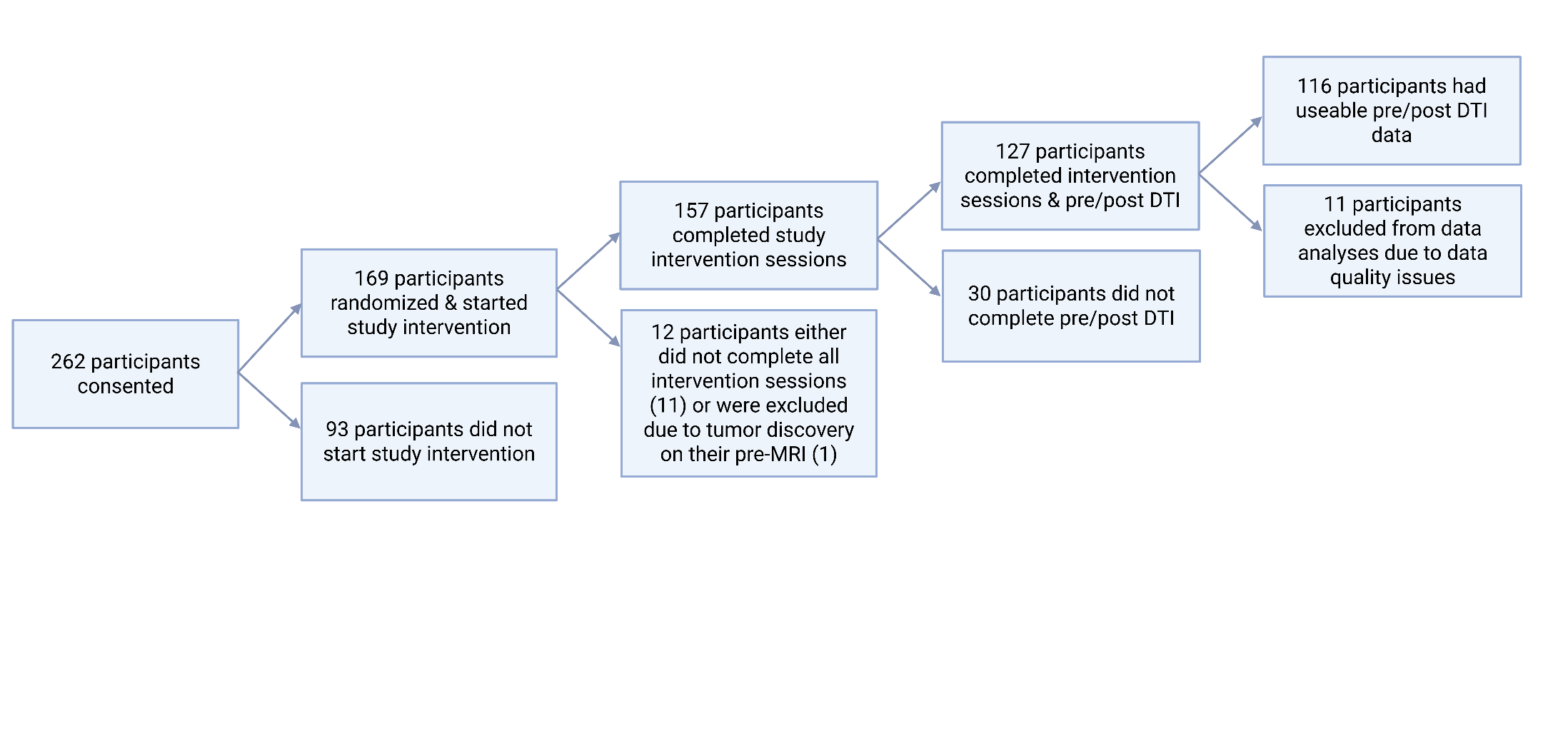
**

**Figure S2.** *Experimental Setup.* Respiration (red waves) assessed using a pneumatic cushion (red waves depicted on monitor); for participants in the vibration-augmented breath-focused mindfulness VABF intervention, vibration output signals are sent at the peak of each respiration wave when exhalation starts (blue starred waves on screen). The vibration output signal is sent to a wearable subwoofer placed on the sternum and secured with an elastic strap (blue). Participants in other mindfulness meditation interventions had an identical configuration, but the vibration feedback device was either inactive (breath-focus only, open awareness) or vibrated in a continuous pattern without breath sync (vibration only).

**
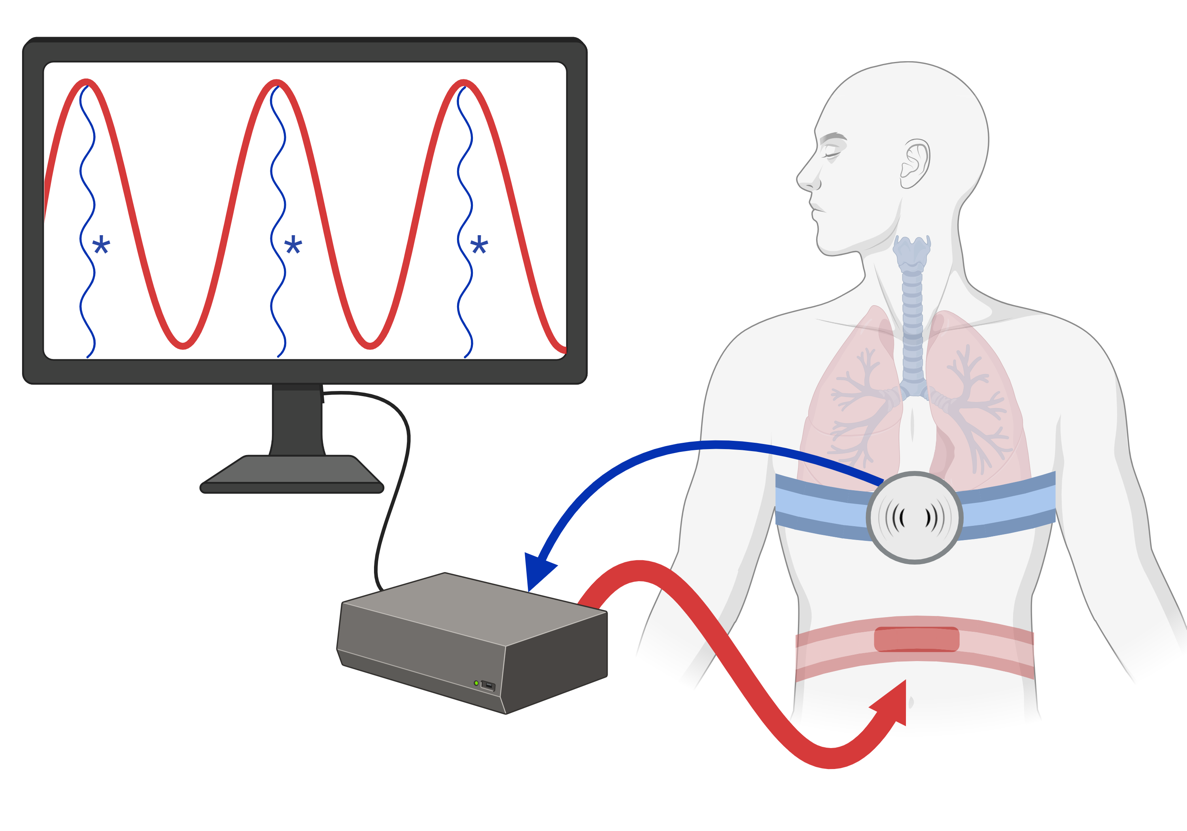
**

**Figure S3.** *White Matter Change and Change in Body Connectedness: Moderation by Intervention Condition*. Moderating effect of vibration on white matter and self-reported body dissociation. Increased NDI associated with decreased body dissociation in vibration groups (red lines) only; no significant relationship between NDI change and body dissociation change was observed in the non-vibration groups (blue lines).


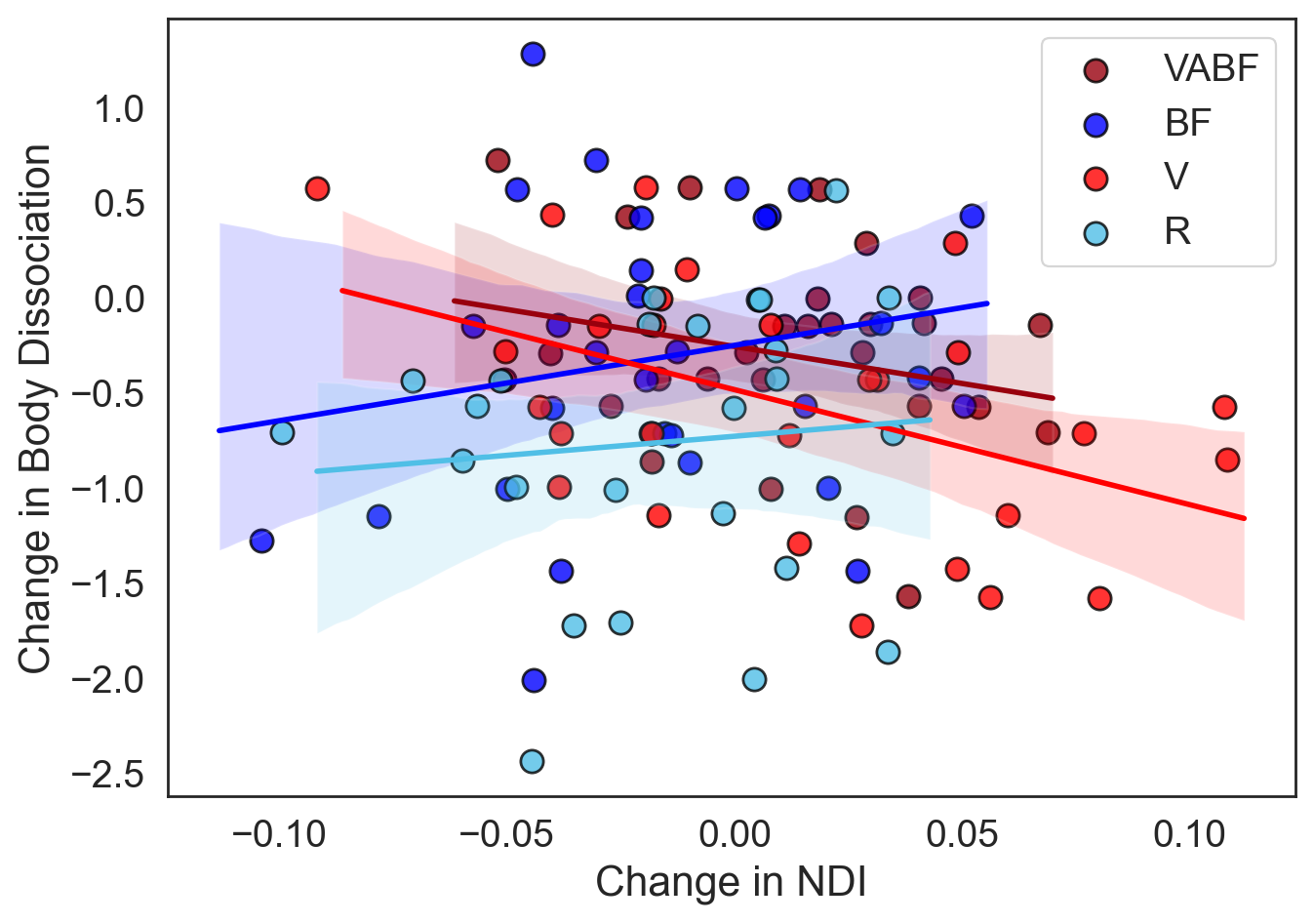


**Figure S4.** *Self-reported Changes in Scale of Body Connectedness Subscales by Vibration Condition*.


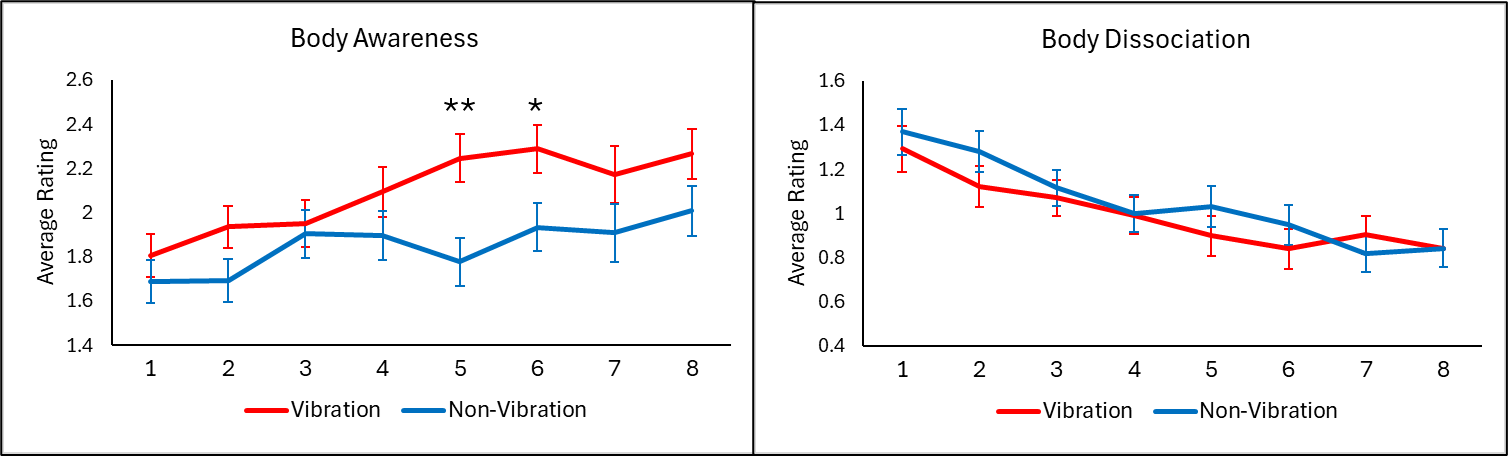
Statistically significant pairwise effect at: **p* < .05, ***p* < .01

**Table S1.** *Medication and Diagnostic Characteristics*

| **Mindfulness Training Group:** | **Vibration (*n*=60)** | **Non-Vibration (*n*=56)** |  |
| --- | --- | --- | --- |
| **Medication** | **% (*n*)** | | **Pearson χ^2^ Tests** |
| Antidepressant (SSRI, SNRI, etc.) | 25.0 (15) | 28.6 (16) | χ^2^=0.2 |
| Anticonvulsant/Mood Stabilizer | 8.3 (5) | 7.1 (4) | χ^2^=0.1 |
| Antipsychotic | 1.7 (1) | 0 | χ^2^=0.9 |
| Benzodiazepine | 8.3 (5) | 7.1 (4) | χ^2^=0.1 |
| Serotonergic Receptor | 1.7 (1) | 3.6 (2) | χ^2^=0.4 |
| Stimulant | 6.7 (4) | 10.7 (6) | χ^2^=0.6 |
| Muscle Relaxant | 5.0 (3) | 1.8 (1) | χ^2^=0.9 |
| Hypnotics | 3.3 (2) | 0 | χ^2^=1.9 |
| Cardiovascular | 8.3 (5) | 10.7 (6) | χ^2^=0.2 |
| Endocrine | 16.7 (10) | 17.9 (10) | χ^2^=0.02 |
| Thyroid Agent | 0 | 1.8 (1) | χ^2^=1.1 |
| **Psychiatric Diagnosis** | **% (*n*)** | | **Pearson χ^2^ Tests** |
| Current Posttraumatic Stress Disorder | 63.3 (38) | 60.7 (34) | χ^2^=0.2 |
| Lifetime Posttraumatic Stress Disorder | 78.3 (47) | 69.6 (39) | χ^2^=1.1 |
| Current Major Depressive Disorder | 38.3 (23) | 35.7 (20) | χ^2^=0.1 |
| Lifetime Major Depressive Disorder | 90.0 (54) | 83.9 (47) | χ^2^=0.9 |
| Bipolar Disorder II | 3.3 (2) | 7.1 (4) | χ^2^=0.9 |
| Mood disorder w/psychotic features | 3.3 (2) | 3.6 (2) | χ^2^=0.01 |
| Current Panic Disorder | 13.3 (8) | 12.5 (7) | χ^2^=0.02 |
| Lifetime Panic Disorder | 30.0 (18) | 32.1 (18) | χ^2^=0.06 |
| Current Agoraphobia | 16.7 (10) | 5.4 (3) | χ^2^=3.6 |
| Current Social Anxiety Disorder | 18.3 (11) | 25.0 (14) | χ^2^=0.8 |
| Current Obsessive-Compulsive Disorder | 15.0 (9) | 16.1 (9) | χ^2^=0.03 |
| Current Generalized Anxiety Disorder | 31.7 (19) | 28.6 (16) | χ^2^=0.1 |
| Current Alcohol Use Disorder | 10.0 (6) | 8.9 (5) | χ^2^=0.04 |
| Lifetime Alcohol Use Disorder | 31.7 (19) | 33.9 (19) | χ^2^=0.07 |
| Current Substance Use Disorder | 13.3 (8) | 14.3 (8) | χ^2^=0.02 |
| Lifetime Substance Use Disorder | 30.0 (18) | 25.0 (14) | χ^2^=0.4 |

| **Table S2.** *Replication Analyses with Tractography Assessing Intervention Effects on Neurite Density Index* | | | | | |
| --- | --- | --- | --- | --- | --- |
| Outcome Measure | Effect | *F*-value | *Degrees of Freedom* | *p*-value | Effect Size (*η_p_*^2^) |
| Left Cerebrospinal Tract NDI | Time | 0.17 | *1 / 109* | *p* = .681 | *η_p_*^2^ = .002 |
|  | **Time x Vibration*** | **7.68** | ***1 / 109*** | ***p* = .007** | ***η_p_*^2^ = .066** |
| Right Cerebrospinal Tract NDI | Time | 0.55 | *1 / 109* | *p* = .459 | *η_p_*^2^ = .005 |
|  | **Time x Vibration*** | **8.88** | ***1 / 109*** | ***p* = .004** | ***η_p_*^2^ = .075** |
| Left Temporal Sections of the Cingulum | Time | 1.84 | *1 / 109* | *p* = .178 | *η_p_*^2^ = .017 |
|  | Time x Vibration | 0.38 | *1 / 109* | *p* = .539 | *η_p_*^2^ = .003 |
| Right Temporal Sections of the Cingulum | Time | 2.23 | *1 / 109* | *p* = .139 | *η_p_*^2^ = .020 |
|  | Time x Vibration | 0.02 | *1 / 109* | *p* = .878 | *η_p_*^2^ < .001 |

* with bolded text indicates significance at *p* < .05.

| **Table S3.** *Self-reported Changes in Scale of Body Connectedness (SBC) Ratings* | | | | | |
| --- | --- | --- | --- | --- | --- |
| Outcome Measure | Effect | *F*-value | *Degrees of Freedom* | *p*-value | Effect Size (*η_p_*^2^) |
| SBC: Body Awareness | **Time*** | **6.41** | ***7 / 100*** | ***p < .001*** | ***η_p_*^2^ *=* .310** |
|  | **Time x Vibration*** | **2.30** | ***7 / 100*** | ***p = .032*** | ***η_p_*^2^ *=* .139** |
| SBC: Body Dissociation | **Time*** | **10.17** | ***7 / 100*** | ***p < .001*** | ***η_p_*^2^ *=* .416** |
|  | Time x Vibration | 0.95 | *7 / 100* | *p = .472* | *η_p_*^2^ *= .062* |

* with bolded text indicates significance at *p* < .05.

| **Table S4.** *Confirmatory ANOVAs Testing with Covariates of Baseline PTSD Symptoms/Diagnosis, Depression Symptoms/Diagnosis, Prior Meditation, and Days Between Final Intervention and Post-scan* | | | | | |
| --- | --- | --- | --- | --- | --- |
| Outcome Measure | Effect | *F*-value | *Degrees of Freedom* | *p*-value | Effect Size (*η_p_*^2^) |
| Left Cerebrospinal Tract NDI  *(PCL-5 Scores*  *as Covariate)* | Time | 0.99 | *1 / 108* | *p* = .320 | *η_p_*^2^ = .009 |
|  | **Time x Vibration*** | **7.31** | ***1 / 108*** | ***p* = .008** | ***η_p_*^2^ = .063** |
|  | Time x PTSD Symptoms | 0.84 | *1 / 108* | *p* = .363 | *η_p_*^2^ = .008 |
| Right Cerebrospinal Tract NDI  *(PCL-5 Scores*  *as Covariate)* | Time | 0.14 | *1 / 108* | *p* = .906 | *η_p_*^2^ < .001 |
|  | **Time x Vibration*** | **8.62** | ***1 / 108*** | ***p* = .004** | ***η_p_*^2^ = .074** |
|  | Time x PTSD Symptoms | 0.15 | *1 / 108* | *p* = .700 | *η_p_*^2^ = .001 |
| Left Cerebrospinal Tract NDI  *(CAPS-5 Diagnosis*  *as Covariate)* | Time | 0.59 | *1 / 105* | *p* = .445 | *η_p_*^2^ = .006 |
|  | **Time x Vibration*** | **8.41** | ***1 / 105*** | ***p* = .005** | ***η_p_*^2^ = .074** |
|  | Time x PTSD Diagnosis | 0.58 | *1 / 105* | *p* = .448 | *η_p_*^2^ = .005 |
| Right Cerebrospinal Tract NDI  *(CAPS-5 Diagnosis*  *as Covariate)* | Time | 0.003 | *1 / 105* | *p* = .958 | *η_p_*^2^ = .000 |
|  | **Time x Vibration*** | **8.88** | ***1 / 105*** | ***p* = .004** | ***η_p_*^2^ = .078** |
|  | Time x PTSD Diagnosis | 0.31 | *1 / 105* | *p* = .578 | *η_p_*^2^ = .003 |
| Left Cerebrospinal Tract NDI  *(PROMIS-D*  *as Covariate)* | Time | 0.59 | *1 / 108* | *p* = .443 | *η_p_*^2^ = .005 |
|  | **Time x Vibration*** | **7.33** | ***1 / 108*** | ***p* = .008** | ***η_p_*^2^ = .064** |
|  | Time x Depression Symptoms | 0.46 | *1 / 108* | *p* = .500 | *η_p_*^2^ = .004 |
| Right Cerebrospinal Tract NDI  *(PROMIS-D*  *as Covariate)* | Time | 0.03 | *1 / 108* | *p* = .875 | *η_p_*^2^ < .001 |
|  | **Time x Vibration*** | **8.72** | ***1 / 108*** | ***p* = .004** | ***η_p_*^2^ = .075** |
|  | Time x Depression Symptoms | 0.01 | *1 / 108* | *p* = .945 | *η_p_*^2^ < .001 |
| Left Cerebrospinal Tract NDI  *(MDE Diagnosis*  *as Covariate)* | Time | 0.38 | *1 / 108* | *p* = .540 | *η_p_*^2^ = .003 |
|  | **Time x Vibration*** | **7.74** | ***1 / 108*** | ***p* = .006** | ***η_p_*^2^ = .067** |
|  | Time x MDE Diagnosis | 0.23 | *1 / 108* | *p* = .633 | *η_p_*^2^ = .002 |
| Right Cerebrospinal Tract NDI  *(MDE Diagnosis*  *as Covariate)* | Time | 0.37 | *1 / 108* | *p* = .542 | *η_p_*^2^ = .003 |
|  | **Time x Vibration*** | **8.76** | ***1 / 108*** | ***p* = .004** | ***η_p_*^2^ = .075** |
|  | Time x MDE Diagnosis | 0.001 | *1 / 108* | *p* = .975 | *η_p_*^2^ < .001 |
| Left Cerebrospinal Tract NDI  *(Prior Meditation*  *as Covariate)* | Time | 0.25 | *1 / 108* | *p* = .621 | *η_p_*^2^ = .002 |
|  | **Time x Vibration*** | **7.64** | ***1 / 108*** | ***p* = .007** | ***η_p_*^2^ = .066** |
|  | Time x Prior Meditation | 0.08 | *1 / 108* | *p* = .782 | *η_p_*^2^ = .001 |
| Right Cerebrospinal Tract NDI  *(Prior Meditation*  *as Covariate)* | Time | 0.07 | *1 / 108* | *p* = .798 | *η_p_*^2^ = .001 |
|  | **Time x Vibration*** | **10.5** | ***1 / 108*** | ***p* = .002** | ***η_p_*^2^ = .089** |
|  | Time x Prior Meditation | 2.57 | *1 / 108* | *p* = .112 | *η_p_*^2^ = .023 |
| Left Cerebrospinal Tract NDI  *(Days Between Last Intervention Session and Post-Scan*  *as Covariate)* | Time | 0.03 | *1 / 108* | *p* = .874 | *η_p_*^2^ < .001 |
|  | **Time x Vibration*** | **7.33** | ***1 / 108*** | ***p* = .008** | ***η_p_*^2^ = .064** |
|  | Time x Days Between | 0.47 | *1 / 108* | *p* = .496 | *η_p_*^2^ = .004 |
| Right Cerebrospinal Tract NDI  *(Days Between Last Intervention Session and Post-Scan*  *as Covariate)* | Time | 0.19 | *1 / 108* | *p* = .664 | *η_p_*^2^ = .002 |
|  | **Time x Vibration*** | **8.82** | ***1 / 108*** | ***p* = .004** | ***η_p_*^2^ = .075** |
|  | Time x Days Between | 0.03 | *1 / 108* | *p* = .872 | *η_p_*^2^ < .001 |

*with bolded text indicates significance at *p* < .05.
